# Anomaly detection over differential preserved privacy in online social networks

**DOI:** 10.1371/journal.pone.0215856

**Published:** 2019-04-25

**Authors:** Randa Aljably, Yuan Tian, Mznah Al-Rodhaan, Abdullah Al-Dhelaan

**Affiliations:** 1 Computer Department, Shaqra University, Druma, Kingdom of Saudi Arabia; 2 Computer Science Department, King Saud University, Riyadh, Kingdom of Saudi Arabia; 3 Nanjing Institute of Technology, Nanjing, China; Wuhan University, CHINA

## Abstract

The massive reach of social networks (SNs) has hidden their potential concerns, primarily those related to information privacy. Users increasingly rely on social networks for more than merely interactions and self-representation. However, social networking environments are not free of risks. Users are often threatened by privacy breaches, unauthorized access to personal information, and leakage of sensitive data. In this paper, we propose a privacy-preserving model that sanitizes the collection of user information from a social network utilizing restricted local differential privacy (LDP) to save synthetic copies of collected data. This model further uses reconstructed data to classify user activity and detect abnormal network behavior. Our experimental results demonstrate that the proposed method achieves high data utility on the basis of improved privacy preservation. Moreover, LDP sanitized data are suitable for use in subsequent analyses, such as anomaly detection. Anomaly detection on the proposed method’s reconstructed data achieves a detection accuracy similar to that on the original data.

## Introduction

Information sharing platforms, such as online social networks (OSNs), have experienced remarkable growth and recognition in recent years. Notably, OSN platforms have direct access to the public and private data of their users [1]. In some cases, these data are shared with other parties to carry out analytical and social research. Although the release of social network data is considered a severe breach of privacy, OSN platforms reassure their users by anonymizing their data before sharing it. Unfortunately, data mining techniques can be used to infer sensitive information from released data. Therefore, it is necessary to sanitize network data before releasing it [2].

Moreover, an increasing number of attacks target personal user information on OSNs [3]. Thus, there is an urgent need for radical improvements in OSN security and privacy measures. Most previous studies on the preserved privacy of published data deal only with relational data and cannot be applied to social network data [4].

Therefore, we have taken the initiative toward preserving privacy in social network data. For each user, we use an activity profile to represent his/her sequence of data. With our model, we aim to investigate the application of LDP to user activity logs. In this model, a data collection server uses a specific partitioning of privacy levels to create Laplacian random noise. However, not all user data are stored in SN repositories; only a predetermined set is selected amongst the salient points representing the data sequence. On the other hand, the data analyzer sub-model reverses these disrupted points to reconstruct the original stored data received from the repositories. Moreover, the data analyzer uses the resulting noisy data to detect anomalous behavior. The data analyzers in the proposed model utilize an extension of the conventional LDP to carry out anomaly detection on reconstructed SN data.

In this paper, our contributions are summarized as follows:

1. We propose a model that protects user privacy in SNs compared with other solutions where sensitive user information is poorly anonymized and can be inferred using data mining. We guarantee a stronger degree of privacy and a lower expected error caused by large data streams. Our privacy preserving model applies Laplace’s probability distribution function (PDF) to generate random noise. To guarantee privacy for each user, this noise is calculated using the user’s data. In addition, it protects not only user profiles but also user activity.
2. We achieve an improved estimation error of % 0.15 over direct LDP estimation [5]. In the direct application of LDP to data, the estimated error is linearly proportional to the size of the data set. Since SN data are highly scalable, the direct LDP approach results in relatively high expected error [6].
3. We conduct experiments on real-world datasets, showing that the proposed framework guarantees privacy and achieves modest overhead performance.
4. We significantly reduce analysis costs. In our algorithm, only selected data are sent to the detection model, which estimates the data required for classification.

This paper is organized as follows. In Section 2, we review related work in LDP privacy preservation in social networks. In Section 3, we formulate the problem and demonstrate a threat model. Section 4 introduces the scientific framework and preliminaries. The proposed model is explained in Section 5, followed by experimental results and a discussion in Section 6. We conclude and present our potential future work in Section 7.

### Threat model

The data repositories in an SN collect and store everything related to its users. Logs may contain the user profiles, activities, and networks of other users and may also store information created without user involvement. Sometimes, the SN shares an anonymized version of this information with other parties for different purposes. Unfortunately, as several recent incidents have demonstrated, releasing even anonymized graphs may lead to the re-identification of individuals within the network [7] and the disclosure of confidential information, which has severe consequences for those involved.

**Fig 1:**
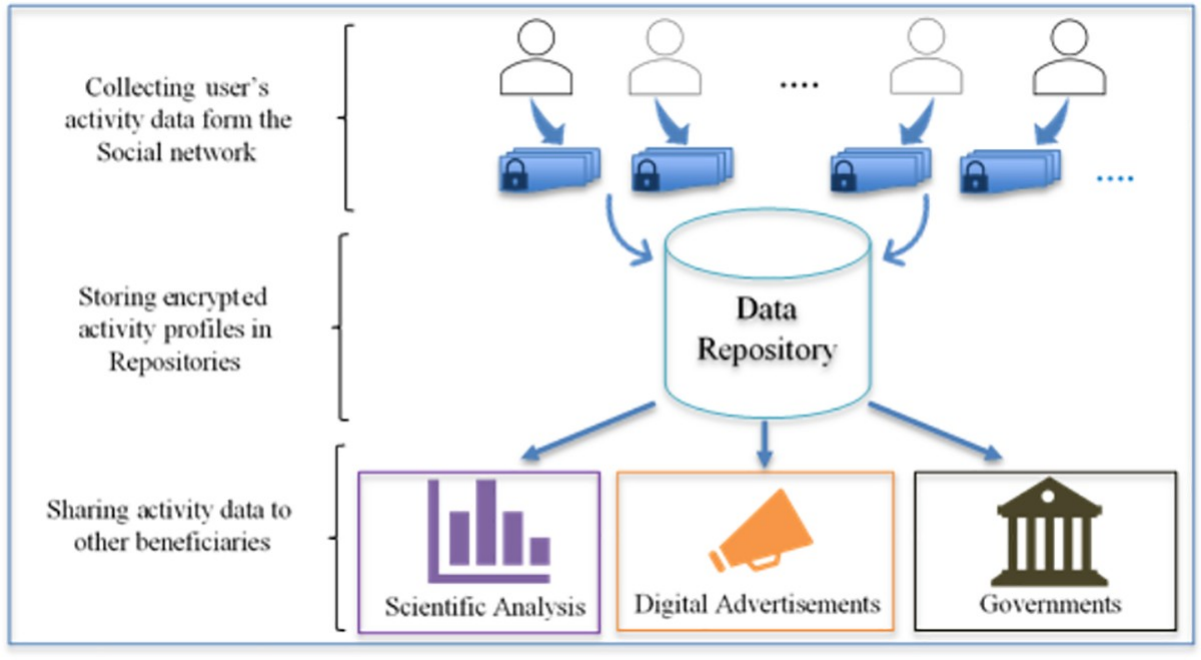
Data collection and sharing in an SN.

Scientific analysis is known to be vulnerable to the identification of individuals and extraction of private data. However, when a specific breach of privacy was tackled with continuous research and proposed solutions, it was shown that data for analysis might be safely released under differential-privacy guarantees [5, 8]. Since privacy preservation in social networks is a relatively new research area, little work has been produced on the application of LDP to user profile data and activity logs. Fig 1 shows the motivational scenario of this research. The SN platform collects a pervasive amount of data and information and immediately stores it in its repositories. The data and information are then shared with the governmental sector under certain agreements. The data may also be shared with analytical parties or even advertising companies to push specifically tailored digital advertisements.

## Related work

Recently, the privacy of social network data has gained increasing attention and concern. Although these types of data are necessary for generating revenue and conducting social research, there is no guarantee that the implemented anonymization techniques will protect users' private information. In this section, we cover the state of the art application of local differential privacy (LDP) in social networks and other fields. LDP in social networks has become an alternative to simple graph anonymization and data aggregation. In one study, out-link privacy was implemented to protect information about individuals that is shared by other users. LDP has even been proposed to solve the non-uniformity problem in two-dimensional multimedia data [8]. Zhou et al. [9] claimed that calculating a standard deviation circle radius defines the divergence of a data grid and allows the dynamic allocation of noise. The results of their proposed model had lower relative errors than similar approaches, such as UG) algorithm. Kim et al. applied LDP to the collection of indoor positioning data and used differentiated data for estimating the density of a specified indoor area [10].

Recently, the application of LDP to crowdsourced data has received substantial attention [11–13]. In this context, LDP is mainly used to collect and build high dimensional data from distributed users [11]. These data are randomized using multi-variate joint distribution estimation on clusters of the dataset, and then the marginal distribution of these clusters is calculated to approximate a new dataset. When the model was tested, the Complexity Reduction Ratio (CRR) reached 0.512. In [12], an online aggregate monitoring framework was designed over infinite streams with a w-event privacy guarantee. The model was combined with a neural network to predict statistical values and test the utility of released data. The resulting mean absolute error (MAE) [0.2–16] and mean relative error (MRE) [0.2–0.6] indicate that the model improved the utility of real-time data publishing. The authors in [13] showed that LDP achieved an %89 assignment success rate in preserving the location of workers in Spatial Crowdsourcing (SC) through the random generation of 1000 work tasks from a dataset of 6100 users.

Privacy preservation in social networks is considered a relatively new research area. The work in [14–18] covers models generally dedicated to preserving privacy in social networks (SNs). The model in [14] tackles the problems of determining data ownership in an SN and the vulnerability of SN metrics to changes in network structure. The authors claim it is necessary to develop an algorithm (such as minimal spanning tree, degree distribution, etc.) to compute results based on differential privacy (DP). Accordingly, modeling the complete 1-neighborhood structure as background knowledge was proven to protect privacy. The model focused on data that could be inferred from neighboring data and provided accurate answers to aggregate queries [15]. In addition to content, the correlation of a SN was investigated in [16]. The described algorithm labels vertexes in the dataset, uses dense clusters to populate an adjacency matrix, and applies a data partition to the matrix to identify dense regions. Lastly, DP is applied to obtain a noisy adjacency matrix. However, LDP has not always been preferred by researchers to preserve the privacy of sensitive attributes in SNs. Cai et al. and Backstrom et al. [17,18] suggest that, although LDP is generally suitable for inherent data, it is not the best choice for preventing inference attacks.

Furthermore, in [19], experiments showed that no LDP algorithm could fully preserve the persistent homology of high dimensional network features or fulfill all network graph metrics. Some proposed solutions for this issue include using Merging Barrels and Consistency Inference [20], deep neural networks with %73 accuracy [21] and neighborhood randomization [22]. An opposing opinion in [23] emphasizes the LDP’s ethical and logistical capacity to protect organic data. The authors demonstrate that LDP can produce a differentially private synthetic dataset to be publicly distributed when combined with other privacy-protecting techniques, such as Ullman’s Private Multiplicative Weights.

Local Differential Privacy Obfuscation (LDPO) is a variation of LDP tailored for IoT. LDPO substitutes homomorphic encryption to distill and aggregate data at edge servers with decreased computational overhead. The model is distributed over devices and both edge and cloud servers and provides an accuracy of %90.45 when using 30 features through feature distillation [24].

In summary, as privacy concerns are being raised ever more frequently, several local differential privacy models have been suggested and proven in many application areas for protecting user privacy from untrusted entities.

## Preliminaries

### Local differential privacy

LDP is a highly reliable and mathematically rigorous privacy standard [25, 26] that injects randomized noise into collected data or query results to hide sensitive details in a dataset. Thus, regardless of the experience level of an attacker, he/she cannot infer any knowledge from differentially elicited data [8, 27].

***Definition (1): (ε−differential privacy)***

Given two statistical datasets, D and $\overset{´}{D}$, which satisfy $|D-\overset{´}{D}|=1$ (Hammingdistance), the randomized function A achieves ε−differential privacy on the condition that

$$e^{-\varepsilon }\leq \frac{\mathrm{Pr}\left(A(D)\in R\right)}{\mathrm{Pr}\left(A(\overset{´}{D})\in R\right)}\leq e^{\varepsilon }.$$ (1)

***Definition (2) laplace mechanism***

Using scientific data analysis, Dwork et al. [28] proposed the Laplace mechanism, which takes as inputs a database (or stream of data) D, function *f*, and privacy parameter ε (privacy budget) and returns the true output of *f* plus some Laplacian noise. This noise is drawn from a Laplace distribution with the probability density function

$$P(x|\lambda )=\frac{1}{2\lambda }e^{-|x|/\lambda }.$$ (2)

where λ is determined by both GS(f) and the desired privacy level ε.

***Theorem 1***: For any function f: $D\to R^{d}$, the mechanism A for any dataset D∈D,

$$A(D)=f(D)+\langle Laplace(\frac{\Delta (f)}{\varepsilon })\rangle^{d},$$ (3)

satisfies ε-differential privacy, where the noise, $Lap\left(\frac{\Delta (f)}{\varepsilon }\right),$ is drawn from a Laplace distribution with a mean of zero and scale of Δ(f)/ε.

### Probability Density Function (PDF)

A random variable has a {\displaystyle {\textrm {Laplace}}(\mu, b)}*Laplace*(*μLaplace*(*μ*,*b*) distribution if its probability density function is

$$f(x|\mu ,b)=\frac{1}{2b}\exp\left(-\frac{|x-\mu }{b}\right).$$ (4)

### Static / Uniform division

Involves the division of privacy level ***ε*** into smaller levels, (*ε*_1_, *ε*_2_, *ε*_3_,……,*ε*_*r*_), such that

$$\varepsilon_{1}=\varepsilon_{2}=\varepsilon_{3}=\ldots \ldots =\varepsilon_{r}=\frac{\varepsilon }{r}.$$ (5)

### Dynamic / Adaptive division of privacy level

To divide the privacy level, a temporal scale must be introduced. Consider three successive SPs in ascending order: $previous\;(t_{s_{h-1}},x_{s_{h-1}}),\;current\;(t_{s_{h}},x_{s_{h}}),$ and $next\;(t_{s_{h+1}},x_{s_{h+1}}).$ Given system parameter α, we calculate the temporal scale *μ*_*h*_ as [29, 30]

$$\mu_{h}=\left(\frac{\left|t_{s_{h}}-t_{s_{h-1}}|+|t_{s_{h}}-t_{s_{h+1}}\right|}{2}\right){}^{\alpha },$$ (6)

and, considering that privacy level *ε* is divided into (*ε*_1_, *ε*_2_, *ε*_3_,……,*ε*_*h*_)

such that *ε* = ∑_1≤*h*≤*r*_ *ε*_*h*_ and *μ*_*sum*_ = ∑_1≤*h*≤*r*_ *μ*_*h*_, we calculate the individual small privacy level as

$$\varepsilon_{h}=\varepsilon \times \frac{\mu_{h}}{\mu_{sum}}.$$ (7)

### Average error rate

The average error rate has been identified as follows.

Given the noisy salient points $S=({\overset{´}{s}}_{1},{\overset{´}{s}}_{2},\ldots ..,{\overset{´}{s}}_{r})$ from r users [29], we can estimate the average values of the original *x*_*n*_ *at time t*_*n*_, which requires averaging all the noisy values of *x*_*n*_, as

$${AVG}_{est}(x_{n})=\frac{1}{r}\times \sum_{{\overset{´}{s}}_{i}\in S}{\overset{´}{x}}_{n}.$$ (8)

### Conjugate Bayesian method [31–33]

The Bayesian probability function is given as

$$P(c|x)=\frac{P(x|c)P(c)}{P(x)},$$ (9)

○ where *P*(*c*|*x*) is the posterior probability, and *P*(*c*|*x*) is the likelihood;
○ *P*(*c*) is the class prior probability;
○ *P*(*x*) is the prediction prior probability.

A discrete random variable *x* is said to have a Poisson distribution with parameter λ > 0, if, for *k* = 0,1,2,…, the probability mass function of X is given by

$$f(k;\lambda )=Pr(x=k)=\frac{\lambda^{k}e^{-\lambda }}{k!}.$$ (10)

For numerical stability, the Poisson probability mass function should be evaluated as

f(k;λ)=exp{klnλ−λ−lnΓ(k+1)}. (11)

Choose *P*_*ij*_ = Poisson (λ_*ij*_) for the unknown rate parameter λ_*ij*_ >0.

Choose a gamma prior for λ_*ij*_ as this ensures that the posterior predictive distribution for a future period is calculable as a simple ratio of Poisson gamma mass functions.

### Applying the Dirichlet process

Given a measurable set *S*, a base probability distribution *H* and a positive real number *α*, the Dirichlet process *DP*(*H*,*α*) is a stochastic process whose sample path is a probability distribution over S. For any measurable finite partition of $S:{(B}_{i}{)}_{i=1}^{n}$, If *X*~*DP*(*H*,*α*), we have

$$\left(X(B_{1})\ldots \ldots X(B_{n}\right))∼Dir(\alpha H(B_{1})\ldots ..H(B_{n})).$$ (12)

The notation *X*~*DP*(*H*,*α*) indicates that the random variable *X* is distributed according to the distribution *DP*(*H*,*α*), i.e., according to a Dirichlet process with parameter base distribution *H* and real number *α* [31, 33].

The Dirichlet distribution of order *K* ≥ 2 with parameters *α*_1_,…..,*a*_*k*_ > 0 has a probability density function with respect to the Lebesgue measure on the Euclidean space *R*^*k* = 1^ given by

$$f(x_{1}\ldots \ldots .,x_{k};\;\alpha_{1}{,\ldots \ldots .\alpha }_{k})=\frac{1}{B(\alpha )}\prod_{i=1}^{k}x_{i}^{\alpha_{i}-1}.$$ (13)

## Proposed approach

**Fig 2:**
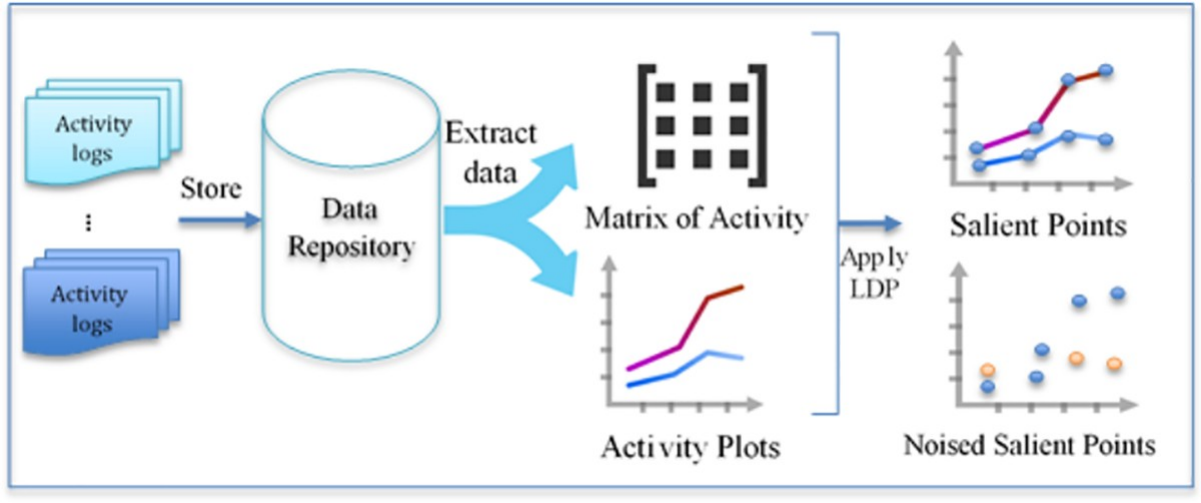
An overview of applying LDP to collected data.

In this section, we describe the proposed scheme for sanitizing SN user activity logs using LDP. We then compare the results of applying anomaly detection to the original and reconstructed data. The model functions on two servers: a data collection server and a data-analyzing server. As shown in Fig 2, the data collection server represents each activity log as a data sequence. In each sequence, we determine specific salient points. After selecting these points, we use the user’s data in addition to other parameters to create random noise. This noise is then added to the data to distort it from its original value. Finally, the data collection server stores it in data repositories.

**Fig 3:**
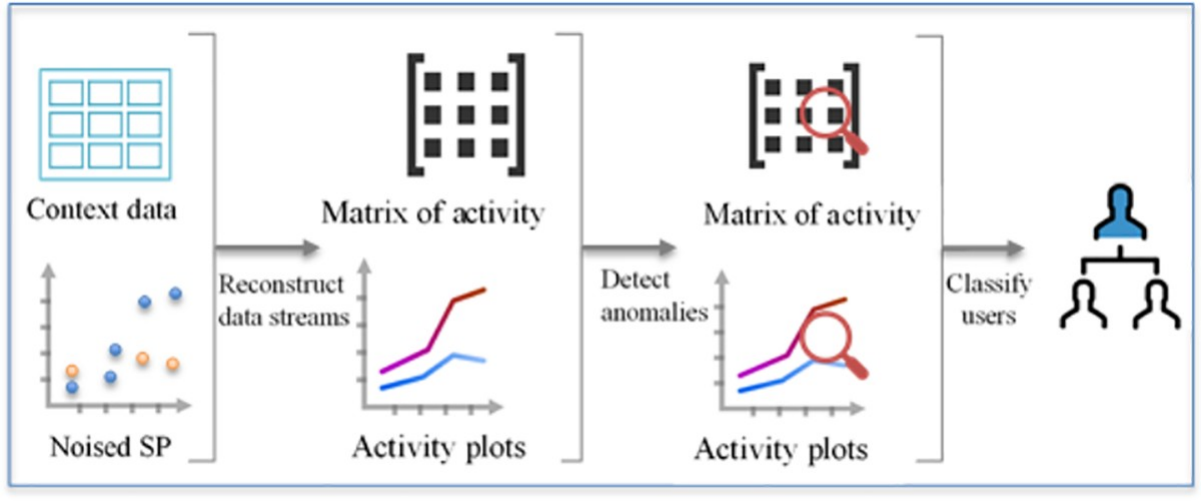
An overview of reconstructing the received data and classifying anomalous behavior.

In contrast, the data-analyzing server retrieves synthetic data from the repositories, reconstructs the original data streams, and searches the user’s activity for abnormal behavior, as demonstrated in Fig 3.

The privacy standard model in the first sub-model avoids high error rates when applying LDP to large datasets. This model essentially groups salient points that represent similar actions (increasing, decreasing, constant) together, then applies LDP to selected points in these groups. Thus, a relatively small number of points are processed.

Users spend significant time on SNs performing all kinds of activities, such as sending messages, posting, liking posts, disliking posts, performing audio or video calling, and so on. If we consider a user’s activity log per single action, it shows active periods vs. non-active periods. If we consider the sending and receiving of messages as an activity, the plot for a particular user’s stream of data increases on days where a greater number of messages are sent and/or received, decreases on days where fewer messages are sent or received and remains constant on idle days.

The model operates in the following order:

Step 1: Calculate the salient points (SPs).

**Fig 4:**
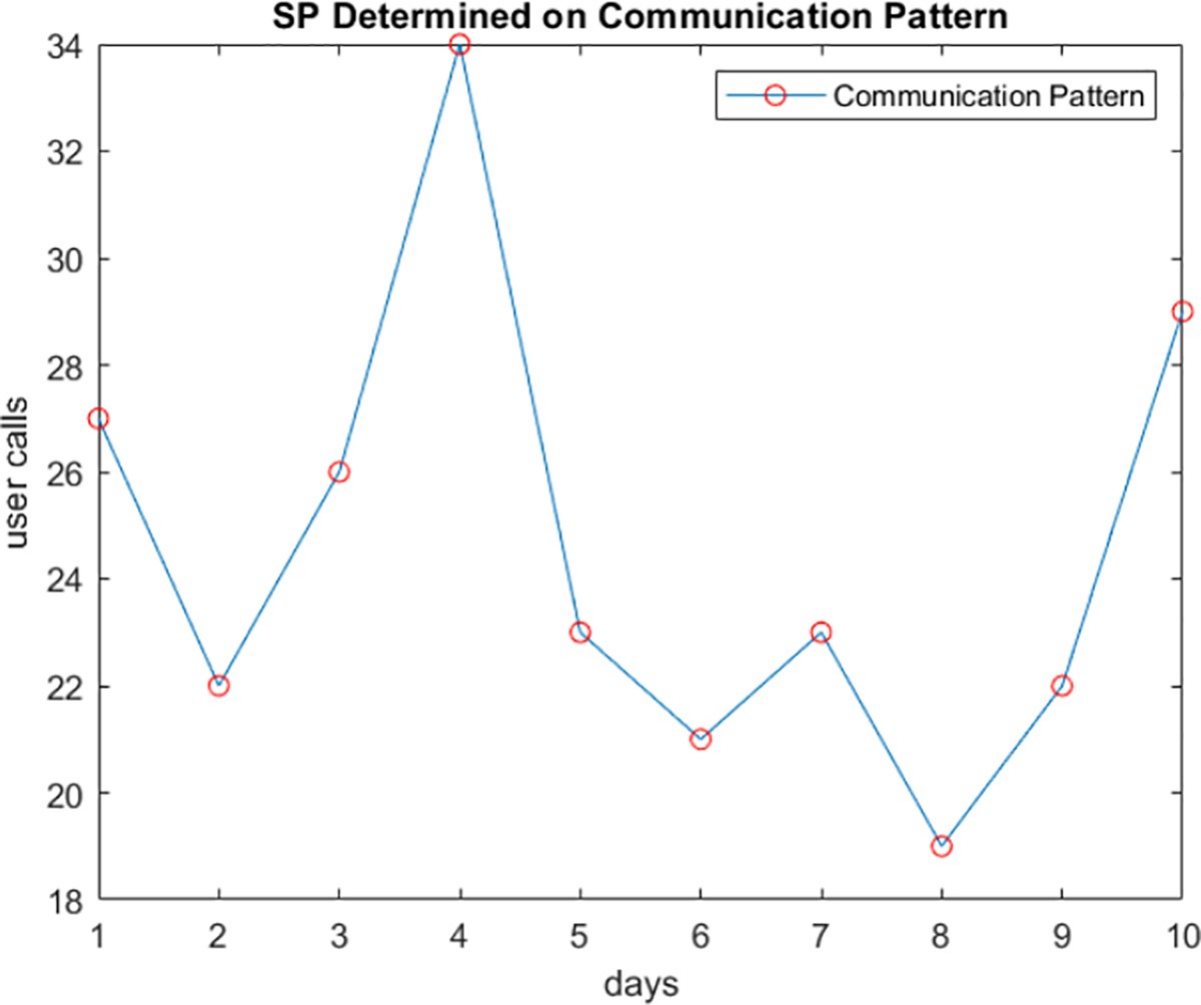
The calling activity of a user.

To obtain the representative points of a user’s data sequence, we take the first order derivative of each value in his/her sequence at a specific timestamp. The user’s sequence is represented as values collected at particular time intervals reflecting increasing, decreasing, or constant activity. In Fig 4, the user’s calling activity is represented as a curve over a ten day time period.

Calculating the first order derivative allows us to determine increasing (derivative >0) and decreasing (derivative <0) periods. We exclude the constant periods where the user’s activity value is the same. As explained in Algorithm 1, we store the points where the derivative of each value is not equal to zero.

Algorithm 1: Pseudo-code for calculating salient points

***Input*:** *S*_*i*_ = ((*t*_1_,*x*_1_),(*t*_2_,*x*_2_),…..,(*t*_*n*_,*x*_*n*_)). ***//*** *Activity stream for a specific action performed by the i*^*th*^ *user*.

**Output**: dS_i_ = ((t_1_,dx_1_),(t_2_,dx_2_),…..,(t_n_,xd_n_))

// Calculate first order derivative

n = size (S_i_)

**For** i← 2: n

      $dx_{i}\leftarrow \frac{{(x}_{i}-x_{i-1})}{{(t}_{i}-t_{i-1})}$

**End For**

// *Exclude* ***x***_***d***_ = **0;**

C_list ← NULL;

**For** i←1: n

      **IF** (dx_i_ ~ = 0)

            add dx_i_ to C_list;

      **End IF**

**End For**

**Fig 5:**
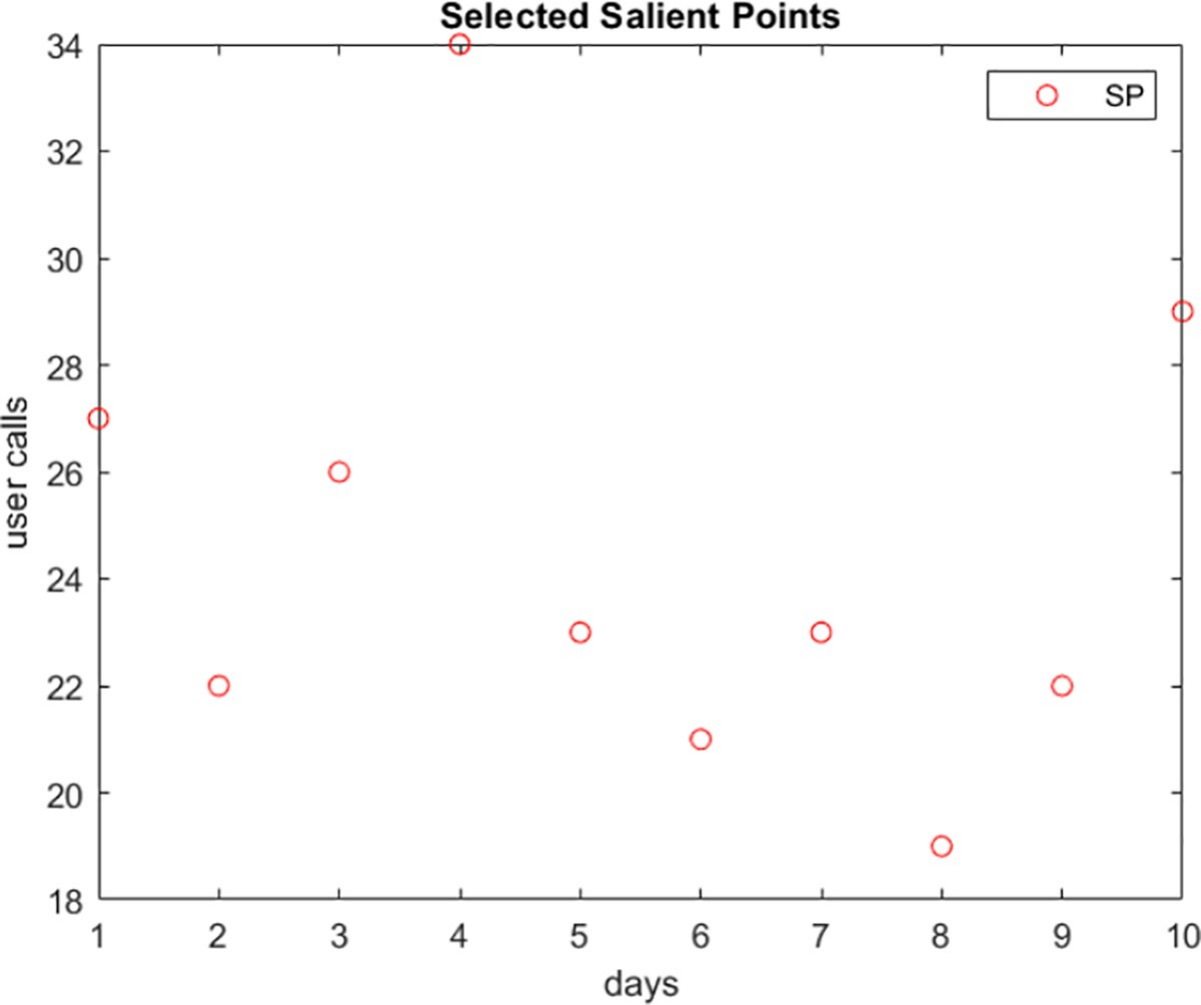
The representative salient points computed from the calling activity in Fig 4.

Fig 5 shows the salient points calculated from the data stream of user calls in Fig 4 (when *dx*_*m*_ ≠ 0). As shown in the Figure, the number of points can be further reduced while maintaining accurate data representation.

Step 2: Reduce the set of salient points to only those indicating the beginning of an increasing or decreasing period following the rule described in Table 1.

**Table 1:** Rule for minimizing salient points.

Condition	Action
If three successive time intervals are all increasing or all decreasing	If the third point is a beginning of an opposite movement (increase after a decrease or decrease after an increase) 1. Add the first two together. 2. Remove the first two points from the list and keep the third.
	Else 1. Add them together. 2.Remove their corresponding points from the List of Points.
Otherwise	1. Keep the corresponding point in the list of points.

In situations where the data sequence is very long (thousands of values), or the data’s time intervals are very small (seconds), the set of salient points will be considerably large and in need of further reduction. To achieve this, any successive points belonging to the same movement can be removed. Therefore, if three successive points belong to an increasing period, we merge their time interval, retaining only the beginning and end of the interval. We continue this reduction process until no two adjacent time intervals have the same movement. Algorithm 2 depicts these steps in detail.

Algorithm 2: Pseudo code for minimizing the number of salient points

***Input*:** *C_list; //First row in C_list is the derivative of the SP*.

*//The second row is the timestamp*.

// Selecting points at the beginning of an ascending or descending period

[~, n] ←size(C_list);

**While** (TRUE)

      interval_min ← ∞;

      **For** h ← 2: n-1

      *// Obtaining the Pre-element*

            Dx_pre ← C_list (1, h-1)

            T_pre ← C_list (2, h-1)

      *// Obtaining the current element*

            Dx_cur← C_list (1, h)

            T_cur← C_list (2, h)

      *// Obtaining the next element*

            Dx_next← C_list (1, h+1)

            T_next← C_list (2, h+1)

      *//Applying the selection condition*

            **IF** (Dx_pre>0 && Dx_cur>0 &&Dx_next >0) OR

                  (Dx_pre<0 && Dx_cur<0 &&Dx_next<0)

                  interval_cur ← |T_cur-T_pre|+|T_cur-T_next|;

                  **IF** (interval_cur < interval_min)

                  interval_min ← interval_cur;

                  t_min ← h

                  **End IF**

            **End IF**

          **End For**

          **IF** (interval_min ← ∞)

                        break

          **End IF**

      Remove element at C_list (1, t_min)

**End while**

Step 3: Calculate the privacy level in uniform and adaptive distributions.

Given the reduced set of salient points, we now partition the privacy level to generate random noise values. This step, uses Algorithm 3. The algorithm divides privacy *ε* into equal levels with each level *ε*_*i*_ satisfying the condition $\varepsilon_{1}=\varepsilon_{2}=\varepsilon_{3}=\ldots \ldots =\varepsilon_{r}=\frac{\varepsilon }{r}$.

Algorithm 3: Pseudo code for uniformly dividing the privacy level

**Inpu**t: n *// Length of the data sequence;*

Epsilon *// Parameter*

Output: *ε*_*i*_

// Uniform partitioning

**For** i←1: n

          *ε*_*i*_← (Epsilon / n);

**End For**

In this step, we calculate a temporal scale for each salient point in the set that controls the privacy level of each point, thus regulating the amount of noise added to it. We use three timestamps representing the current, previous and next SP. We then calculate the temporal sum for all SPs in the sequence of a specific user. Then, we divide the privacy level based on this temporal sum. Algorithm 4 shows the steps of this procedure.

Algorithm 4: Pseudo code for the adaptive division of privacy level

***Input*:**

Selected_SP *// List of selected salient points*

Epsilon *// Parameter*

Alpha *// System parameter*

Beta *// Parameter*

***Output*:** Privacy level for each timestamp *ε*_*i*_.

// Calculating Temporal scale $\mu_{h}=\left(\frac{\left|t_{s_{h}}-t_{s_{h-1}}|+|t_{s_{h}}-t_{s_{h+1}}\right|}{2}\right){}^{\alpha }$

[m, n] ←size (Selected_SP);

**For** i←2: n-1

          Uniform_Up ←|Selected_SP(i)-Selected_SP(i-1) |

                    +

                    |Selected_SP (2, i)-Selected_SP (2, i+1) |

          Fraction ← Uniform_Up/2;

          Temporal_scale(i) ← (Fraction)^Alpha^

**End For**

// The last element in the selected points does not have ‘next’

          Uniform_Up ← |Selected_SP (2, n)-Selected_SP (2, n-1) |

          Fraction ← Uniform_Up/2;

          Temporal_scale(n) ← (Fraction)^Alpha^;

// *Calculating the Temporal sum μ*_*sum*_ = ∑_1≤*h*≤*r*_ *μ*_*h*_

temporal_sum←0

**For** i←1: n

          temporal_sum ← temporal_sum + Temporal_scale(i)

**End For**

// Calculating the privacy level $\varepsilon_{h}=\varepsilon \times \frac{\mu_{h}}{\mu_{sum}}$

**For** i←1: n

    *ε*_*i*_ = Epsilon. ($\frac{Temporal\_scale(i)}{temporal\_sum}$);

**End For**

Step 4: Add Laplacian noise to the selected salient points.

If we consider the list of salient points ${SP}_{i}=\left((t_{s_{1}},x_{s_{1}}),(t_{s_{2}},x_{s_{2}}),\ldots ..,(t_{s_{r}},x_{s_{r}})\right)$, we can obtain the noisy salient points $S{\overset{´}{P}}_{i}=\left((t_{s_{1}},{\overset{´}{x}}_{s_{1}}),(t_{s_{2}},{\overset{´}{x}}_{s_{2}}),\ldots ..,(t_{s_{r}},{\overset{´}{x}}_{s_{r}})\right)$, where ${\overset{´}{x}}_{s_{h}}$ is obtained using the probability distribution function (PDF) of the Laplacian distribution

$${\overset{´}{x}}_{si}=x_{si}+Lap(\frac{\Delta s}{\varepsilon_{i}}).$$ (14)

The Laplacian generated noise depends on the privacy level. Therefore, using a uniform distribution generates noise different from adaptive noise. The higher the value of the privacy level, the higher the generated noise is. Therefore, it differs from one user to another. Knowing that the Laplace distribution performs a simple translation, it perfectly fits with the definition of differential privacy. The steps are shown in Algorithm 5. Since uniform privacy levels are the same, the same PDF generates the noise, whereas, in adaptive privacy partitioning, different PDFs are used to create the noise for each SP. Each different PDF incorporates a different privacy level due to dynamic partitioning, and the PDFs’ are independently calculated for each SP.

Algorithm 5: Calculating Laplacian noise

***Input*:**

Selected_SP [] *// List of Selected Salient points of length n*.

S_max *// Maximum value of the data stream*.

S_min *// Minimum value of the data stream*

Mean_Mu; *// Mean variable for the PDF function*.

Scale_b; *// Standard Deviation for the PDF function*

Uniform_privacy [] *// List of uniform privacy levels for each SP*.

Adaptive_privacy [] *// List of adaptive privacy level for each SP*.

***Output*:**

Uniform_Noisy_stream *// List of distorted SPs using a uniform privacy distribution*.

Adaptive_Noisy_stream *// List of distorted SPs using an adaptive privacy distribution*.

Delta_s = s_max-s_min; *// Data sensitivity (low sensitivity causes higher noise values*)

[m, n]←size(Selected_SP);

**For** i←1: n

    Uniform_Up ← Delta_s/Uniform_privacy(i) // *Delta over uniform epsilon*

    Adaptive_Up ← Delta_s/ Adaptive_privacy(i) // *Delta over adaptive epsilon*

    Uniform_Noise ← pdf ('Normal’, Uniform_Up, Mean_Mue, Scale_b)

    Adaptive_Noise ← pdf ('Normal', Adaptive_Up, Mean_Mue, Scale_b)

    Uniform_Noisy_stream (i) ← Selected_SP(i)+ Uniform_Noise

    Adaptive_Noisy_stream (i) ← Selected_SP(i)+ Adaptive_Noise

**End For**

Step 3: Store the ‘noised’ SP for analytical or other purposes. The repositories contain a sanitized representation of the SP with no indication of the original data.

Step 4: The requesting sub-model receives the noised SP and attempts to reconstruct the stream of data using linear estimation, as explained in the preliminaries section. The sub-model uses the linear equation of a straight line to draw segments between every two points. The general equation is

y=ax+b, (15)

where *a* is the slope of the line, represented as the ($\frac{change\;in\;the\;y-value}{change\;in\;the\;x\;value}$). The y-intercept parameter b is the intersection point between the linear line and the y-axis, which is represented as

$$b={\overset{´}{x}}_{si}-a.t_{si}.$$ (16)

In our case, the slope is calculated as $\frac{change\;in\;noisy\;points}{change\;in\;timestamps}$. Algorithm 6 shows the code steps.

Algorithm 6: Reconstructing the original data stream using linear estimation

**Input**: Uniform_Noisy_stream containing a sequence of the noised SP and a sequence of timestamps for each noised SP.

**Output:** SP *// List of the reconstructed SP*.

[~, n] ← size (Uniform_Noisy_stream);

**For** i←1: n-1

        a(i) ← $\frac{Uniform\_Noisy\_stream(1,i+1)-Uniform\_Noisy\_stream(1,i)}{Uniform\_Noisy\_stream(2,i+1)-Uniform\_Noisy\_stream(2,i)}$

        b(i) ← Uniform_Noisy_stream (1, i)- a(i). Uniform_Noisy_stream (2, i);

**End For**

        a(n) ← Uniform_Noisy_stream (1, n)/Noisy_stream (2, n);

        b(n) ← Uniform_Noisy_stream (1, n)- a(n). Noisy_stream (2, n);

// Reconstructing the original SP

**For** i ← 1: n

SP(i) ← (a(i). Uniform_Noisy_stream (1, i)) + b(i);

**End For**

Step 5: For the activity dataset, the anomaly detection sub-model extracts the number of communications between pairs of nodes as a Bayesian counting process [31] and represents the number of interactions as weights assigned to communicating nodes in the network. The anomaly detection sub-model then applies Bernoulli, Markov chain and Dirichlet processes to find the nonparametric Bayesian inference.

Step 6: Perform individual-based analysis. In this step, we assume *N*_*ij*_(*t*) to be the adjacency of node *i* to node *j* at time *t*. The increments determine the out-degree and in-degree of node *i*, and we represent the number of outgoing nodes as

$$N_{i.}(t)=\sum_{j\neq i}N_{ij}(t),$$ (17)

while the incoming communications over time for individual i are represented as

$$N_{.i}(t)=\sum_{j\neq i}N_{ji}(t).$$ (18)

We then calculate the total activity by finding the degree sum of the network over time N_.._(t).

Step 6: A sample of size *n* is selected from the population. The random variable of interest, *X*, is the number of anomalous individuals in the sample, while *M* is the number of anomalous individuals in the population, and *N* is the set of communicating individuals

P(X=x)=h(x;n,M,n)=(Mx)(N−Mn−x)(Nn). (19)

## Experimental setup

In this section, we describe the simulation process, including the dataset, parameters, and evaluation metrics. We explain the setup and discuss the results in the second sub-section.

**Fig 6:**
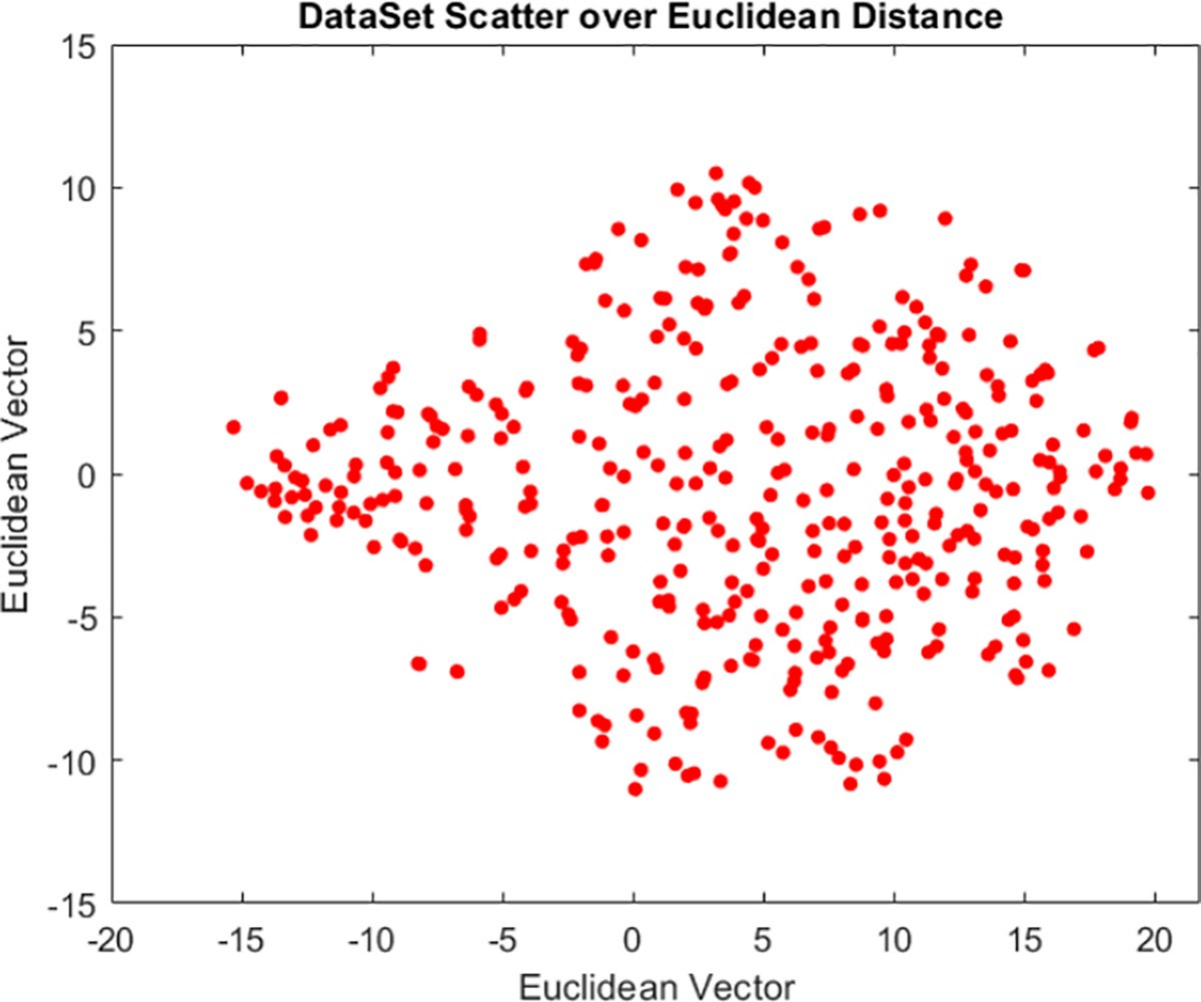
Dataset visualization using MATLAB’s T-SNE function for dimension reduction over Euclidean distance metrics.

We conducted our experiments by applying the LDP privacy preservation distribution to a set of user activity sequences. The data sequence was adapted from the VAST Challenge 2008 [32]. The LDP model was applied to simulated cell phone data from the Mini Challenge on social network analysis. Data were collected from 400 individuals located at 30 locations in the network over a period of ten days. Fig 6 shows a simple visualization of the data after projecting the high dimensional communication log into low-dimensional points.

**Fig 7:**
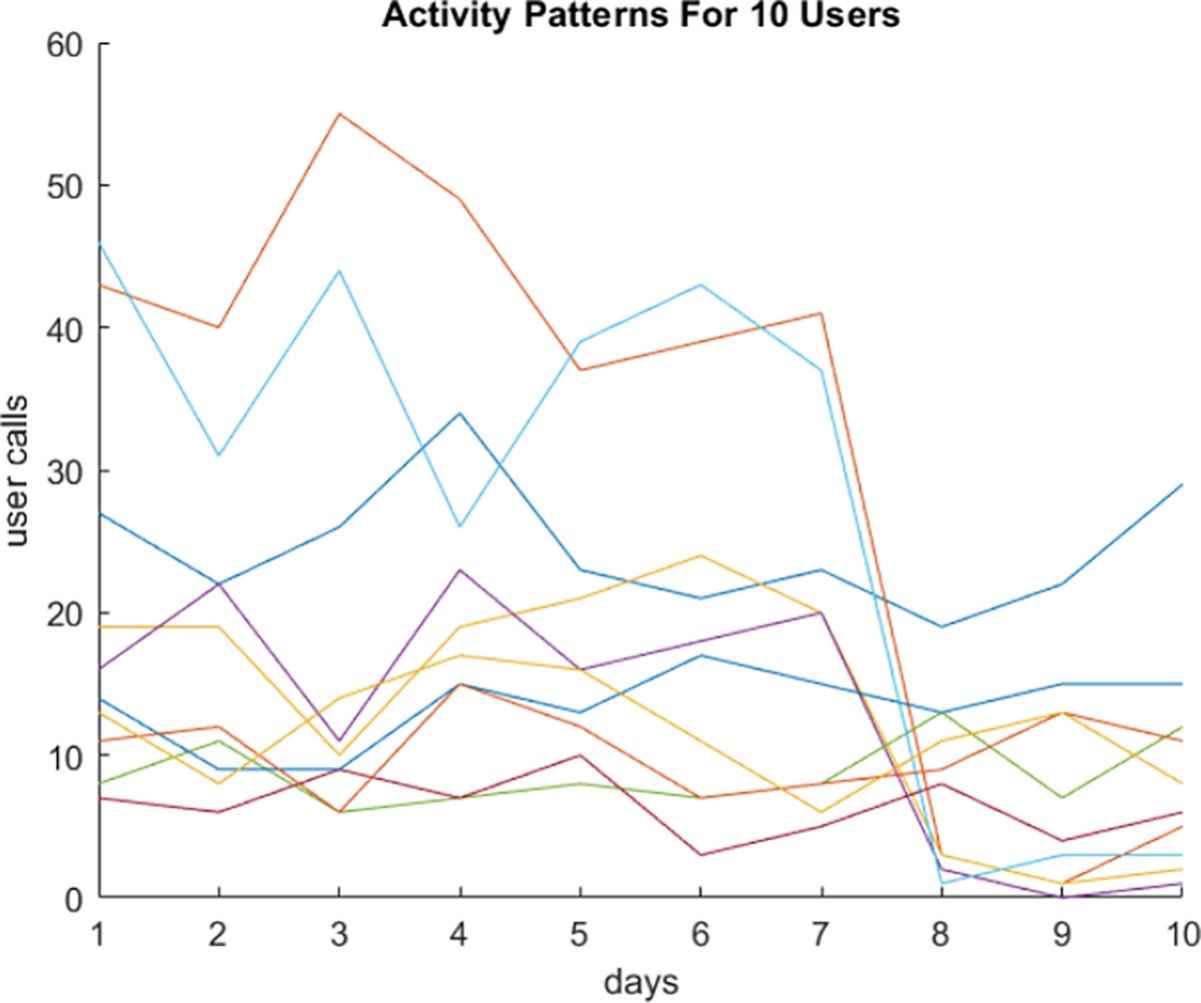
Plotting the activity of 10 users in the dataset.

Each data stream represents a user’s calling activity over ten days. Each day represents a timestamp. Fig 7 illustrates the data sequences (streams) of ten users.

## Results and discussion

**Fig 8:**
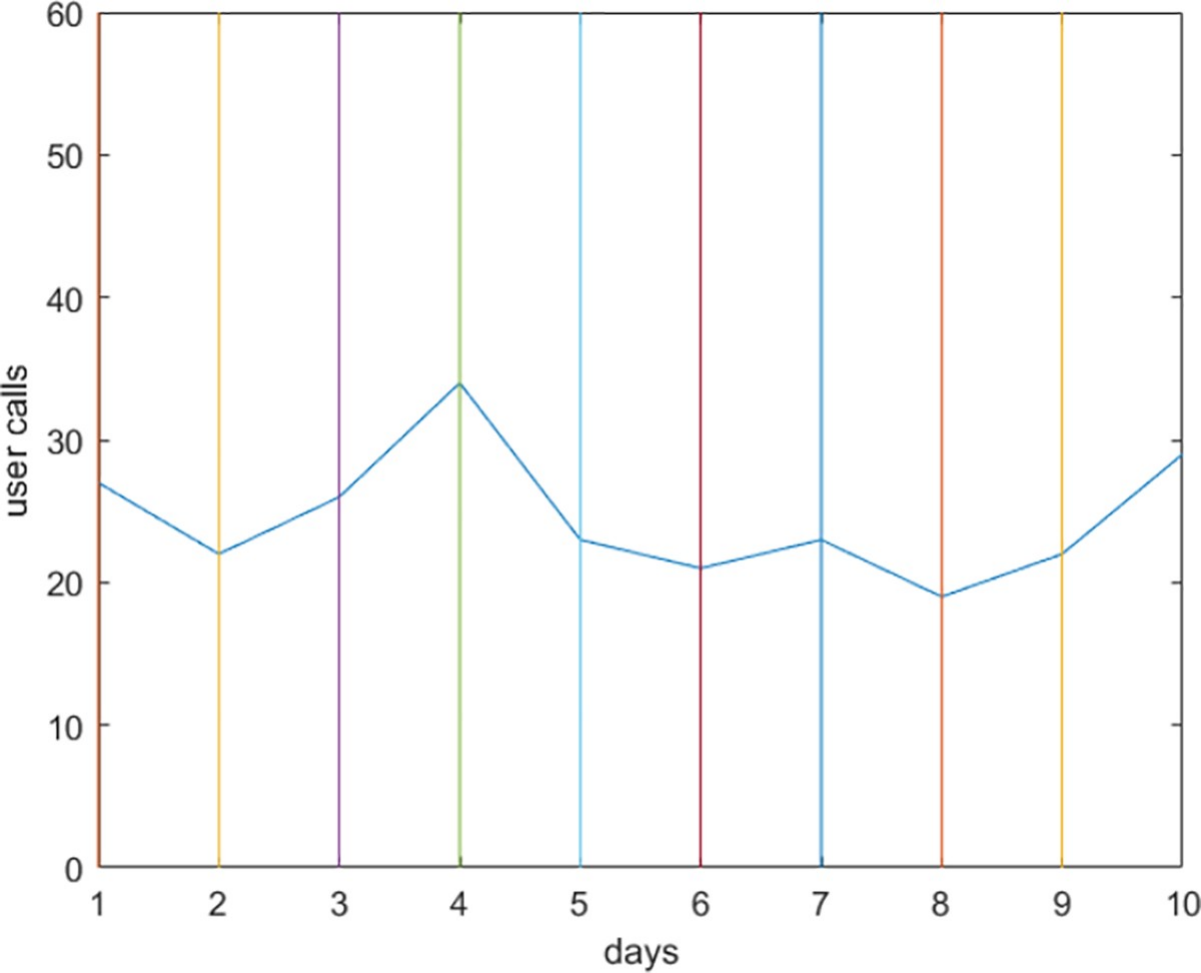
Selecting an SP to represent non-zero derivative periods. All SPs are selected on the curve.

**Fig 9:**
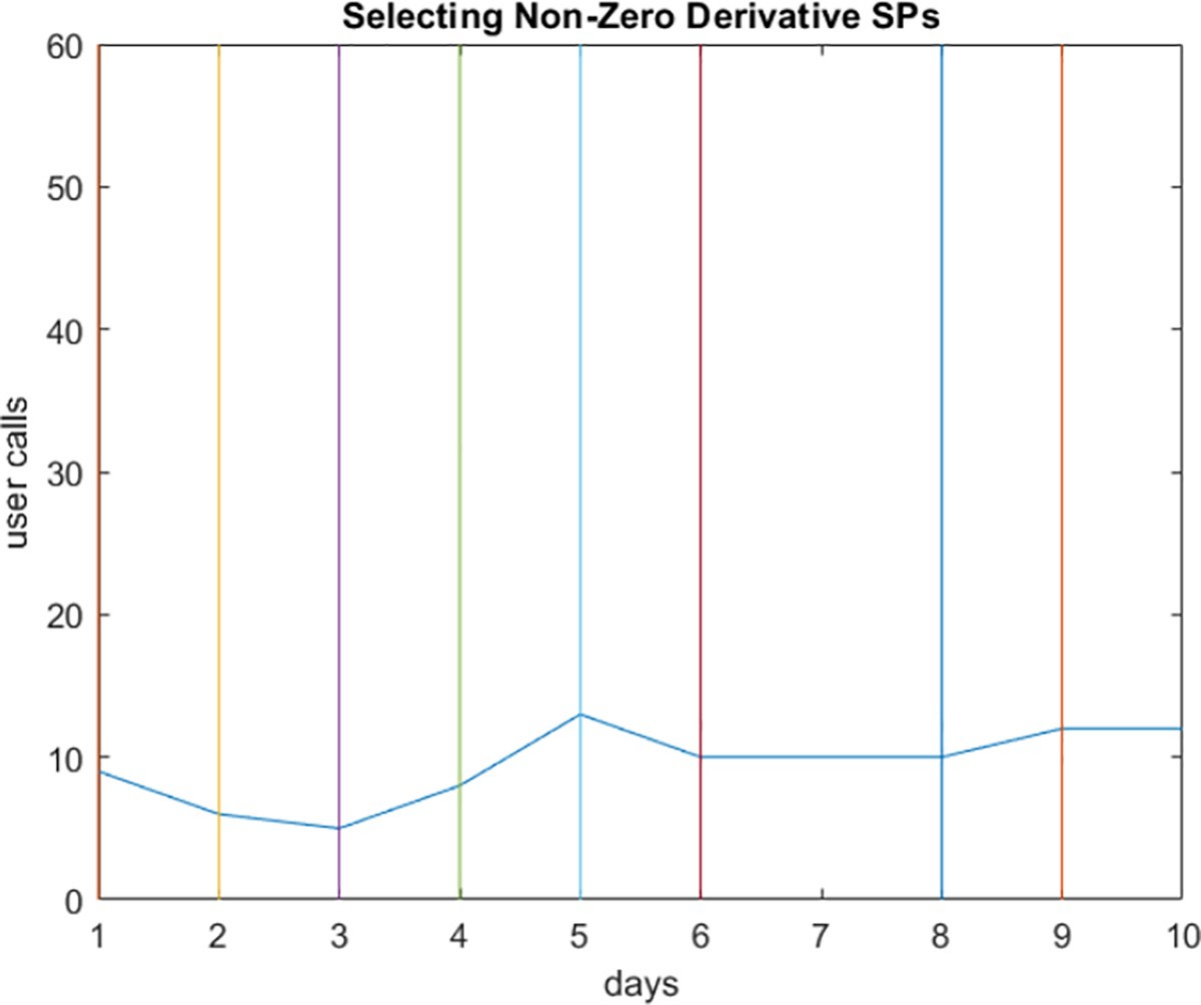
Selecting an SP to represent non-zero derivative periods. The SP for timestamp 7 was (*dx*_*i*_ = 0).

We applied the steps explained in the proposed approach in Section 5. We first determined the salient points in each user’s data stream. The user’s activity in Fig 8 does not contain a constant period (having the same number of calls), so all points are selected. However, the user in Fig 9 makes the same number of calls on the sixth and seventh days (*dx*_*i*_ = 0). Since the first order derivative for timestamp 7 is zero, the salient point at this timestamp is removed. The same applies to timestamp 8; however, since it represents the beginning of a decreasing period, it is retained. The colored lines parallel to the y-axis represent the timestamps, and the intersection point between each line and the curve is a salient point.

**Fig 10:**
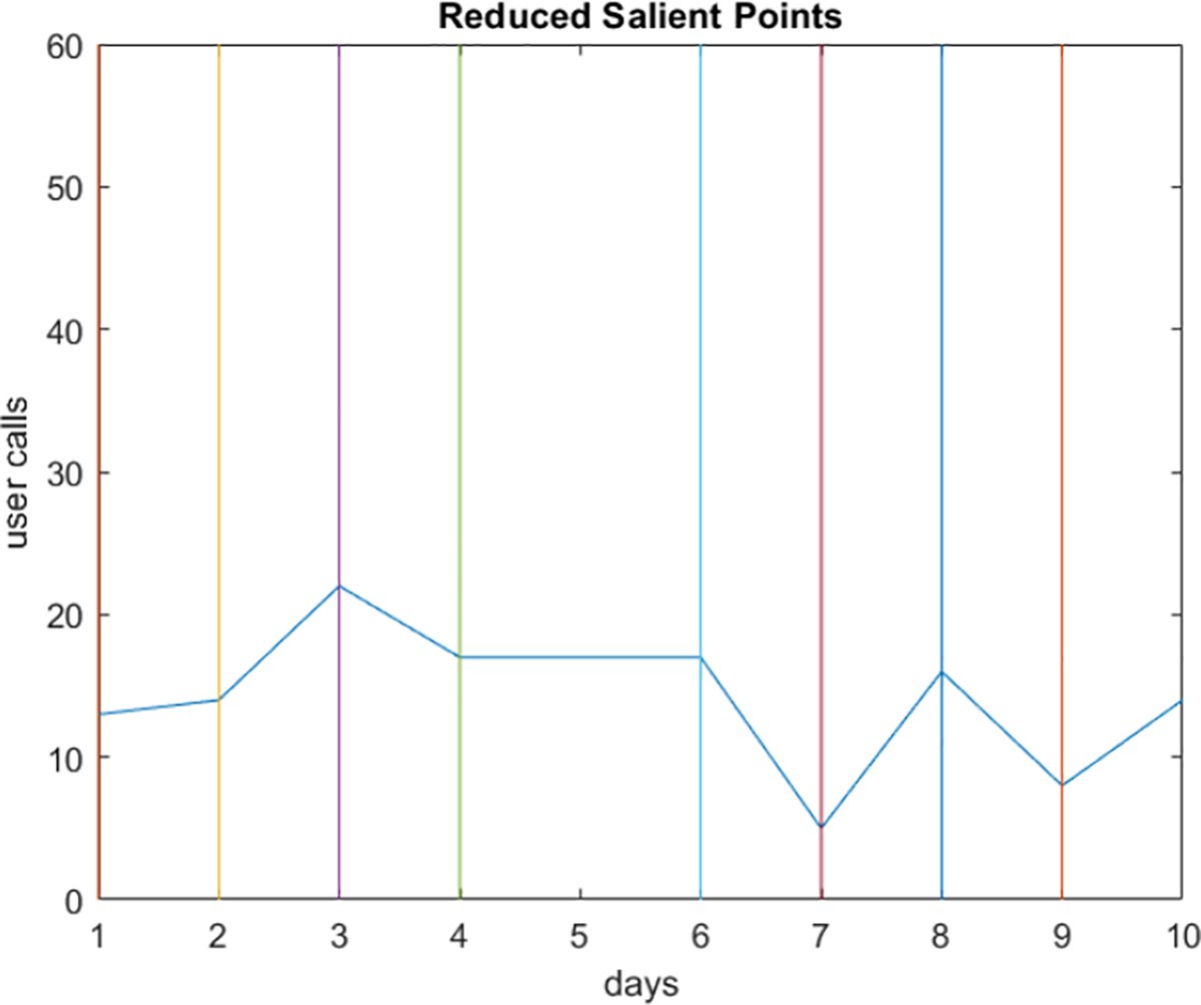
Reduced SPs for a user communication pattern.

**Fig 11:**
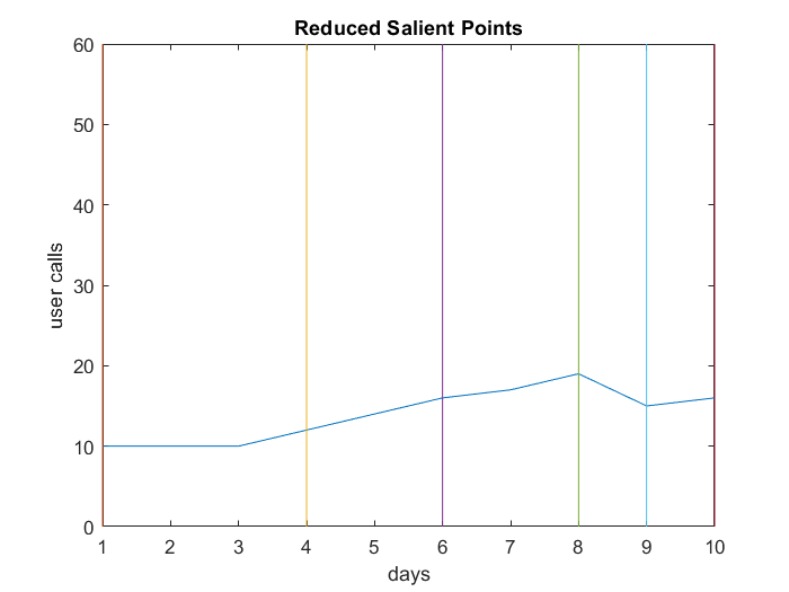
Another example of reduced SPs.

The next step is to reduce the salient points to represent points at the beginning of an increasing or decreasing period. Figs 10 and 11 show the reduced sets of two different users. The user in Fig 10 does not have consecutive intervals with all increasing or decreasing values, as the minimum interval is 2. The same scenario for the other user is shown in Fig 11.

After reducing the set of salient points, we calculate the individual privacy level for each point using uniform or adaptive division and assign Epsilon values of 2 and 5. The Epsilon value is used to generate the random noise applied to the reduced set. We use mean *μ* = 0.8 *scale b* = 0.2 for the PDF function, and noise is added to each point in different data-set sizes: 50, 100, 200 and 400 users.

**Fig 12:**
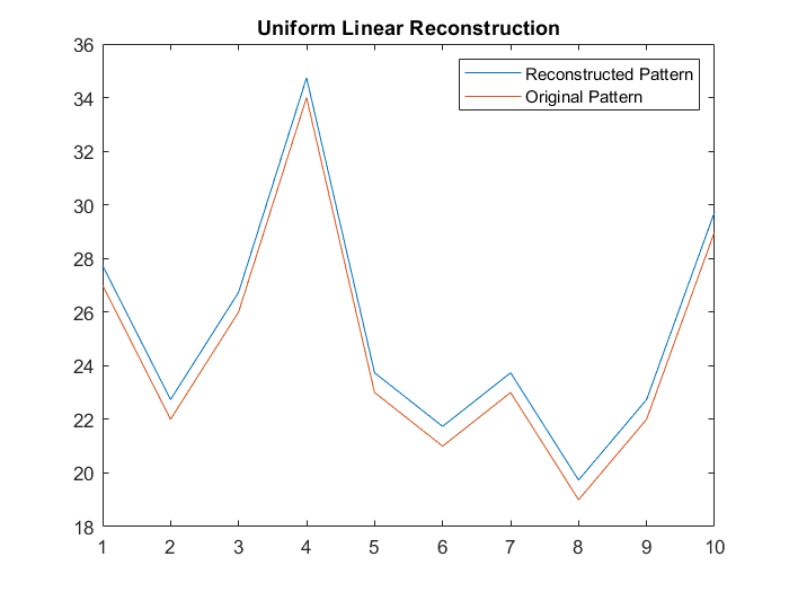
Plotting original and reconstructed curves in a uniform privacy distribution.

**Fig 13:**
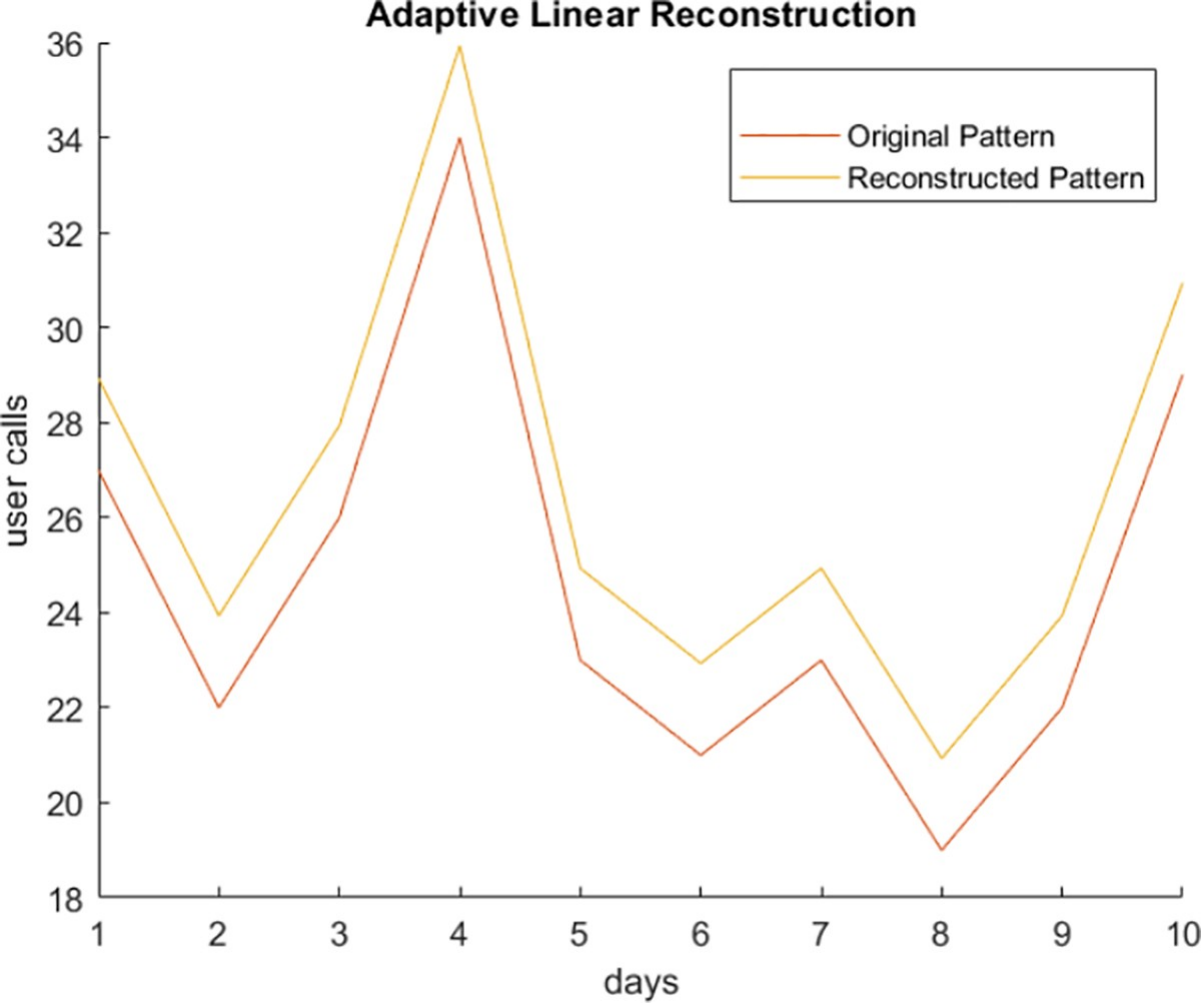
Plotting original and reconstructed curves in adaptive privacy distribution (low noise).

Having created and stored the synthetic data on the data collection server, we next demonstrate the reconstruction process using linear estimation. Figs 12 and 13 show original and reconstructed data streams. In Fig 12, the red curve represents original user activity, and the blue curve represents the activity generated for uniform privacy levels. In Fig 13, the red curve represents the original data stream, and the yellow curve is the linear reconstruction for adaptive distributed privacy levels. Note that the reconstruction of data preserves the structure of the activity pattern, this is very important for anomaly detection.

**Fig 14:**
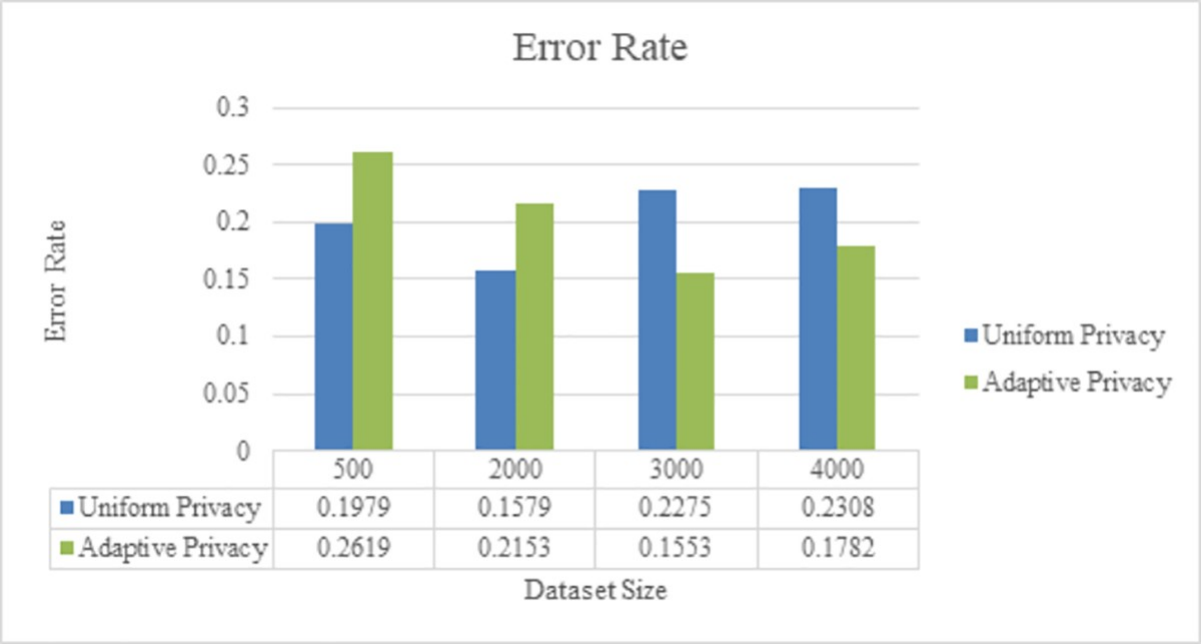
Varying data size (*ε* = 2).

We calculate the error rate for the combination of uniform-privacy division with linear estimation and adaptive-privacy division regenerated using linear estimation. Fig 14 plots the average error rate for the two different approaches on various data sizes. The error rate equation is

$$e=\frac{1}{seq\_length}\times \sum_{time_{stamp}=1}^{n}\frac{Avg(x_{d})-Avg({\overset{´}{x}}_{d})}{Avg(x_{d})}.$$ (20)

where *Avg*(*x*_*d*_) is the average of the actual values in the data stream for timestamp *d*, and $Avg({\overset{´}{x}}_{d})$ is the average of the reconstructed values of the data stream for the same timestamp.

We next apply the Bayesian anomaly detection technique to the reconstructed stream of users. In this experiment, we detect outliers with respect to the duration of calls between individuals. The duration variables are treated in the same manner as the calling activity described earlier. During the first analysis phase, the model checks all 30 locations for anomalous users to apply the multinomial model with the sequential Dirichlet process model with an uninformative negative binomial base measure [7].

**Fig 15:**
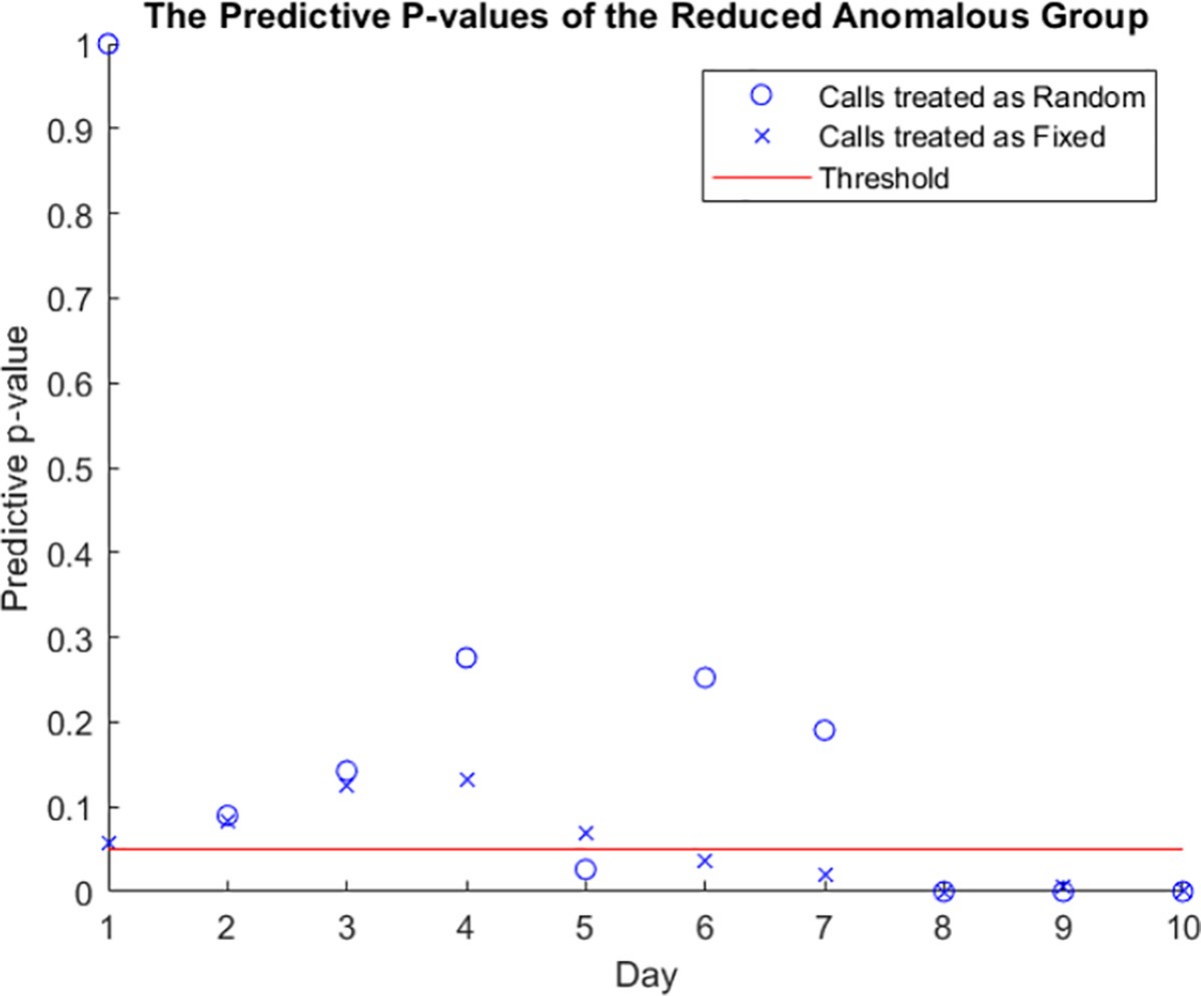
The p-values of the anomalous nodes under the multinomial model.

We apply the Bernoulli process and Markov chain to all network users, with mean values of [0.63, 0.48] using a threshold of 0.05, to obtain a better understanding of the messaging patterns and their variability. This phase extracts the predictive P-values of the users from their communication patterns, as shown in Fig 15. The detection phase of the reconstructed data is the same as that of the original data. The same users have predictive p-values below the threshold and are flagged by the detection sub-model, which implies that the application of LDP to preserve data privacy succeeds in sanitizing the data. In addition, the data structure is maintained for further use by the anomaly detection sub-model.

**Fig 16:**
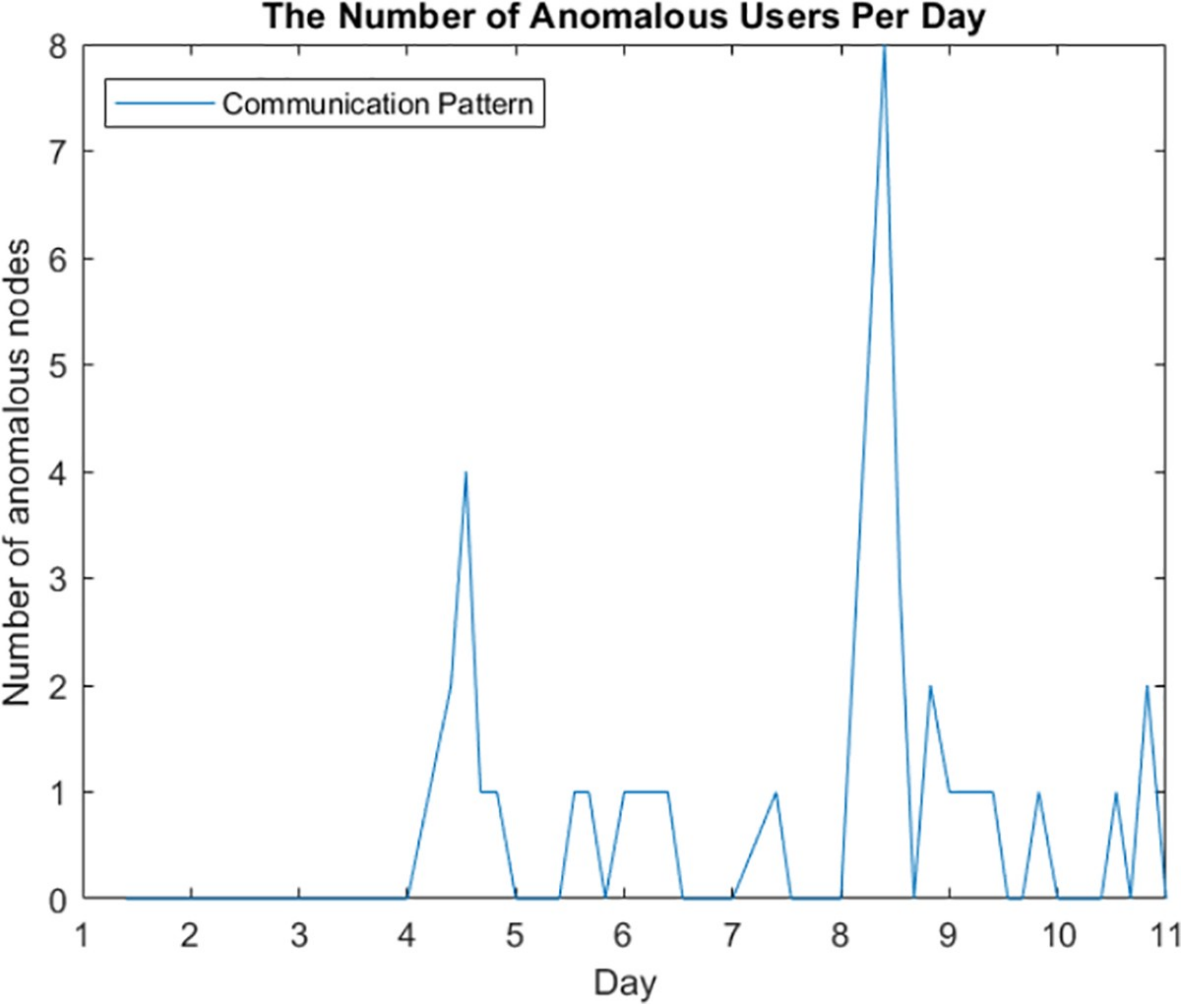
Number of anomalous nodes in each daily time interval under a Markov Bernoulli model.

In Fig 16, the abnormal activities peak on the eighth day, the same day the original activities peak, suggesting that the reconstructed data do not lower the performance of subsequent analyses, which can incorporate all the data into real-time anomaly detection.

As seen in the simulation results in Fig 16, the proposed model improves the estimation error while being applied to large-scale data. The model conducts anomaly detection on a subset of the data without disclosing the actual values, which guarantees privacy and reduces the cost of further analyses.

## Conclusion and future work

In this paper, we presented a model for privacy preservation in social networks. The model sanitizes the collected data and sensitive information of SN users using LDP and then attempts to reconstruct the original sequences and perform analyses using sets of selected salient points. We conserve the social structure of each user’s communication pattern. The error rate of the estimated data compared to the original data is acceptable for large datasets with small time-intervals. Our simulation results show that conducting anomaly detection on synthetic data results in determining the same anomalous users and activities as those in the original data. In the future, we plan to extend the proposed privacy model to include estimating noisy data with non-linear approximation.
